# Individual differences in sustainable working life: genetic and environmental contributions

**DOI:** 10.1093/eurpub/ckac129.440

**Published:** 2022-10-25

**Authors:** P Svedberg, J Narusyte, A Ropponen

**Affiliations:** 1 Divsion Insurance Medicine, Clinical Neuroscience, Karolinska Institutet, Stockholm, Sweden; 2 Center for Epidemiology and Community Medicine, Stockholm County Council, Stockholm, Sweden; 3 Center of Work Ability and Working Careers, Finnish Institute of Occupational Health, Helsinki, Finland

## Abstract

**Background:**

Previous research of aetiology of interruptions in working life have shown that both genetic and environmental factors contribute to individual differences in sickness absence (SA) and disability pension (DP). However, we still lack knowledge about etiological factors contributing to sustainable working life. The aim was to study the importance of genetics, shared (mainly childhood) environmental factors, and individual (unique) environmental factors for remaining in the work force over the life-course, i.e., having a sustainable working life.

**Methods:**

The study population include 108,275 twin individuals born 1930-1990 (53% women) with comprehensive national register data on social security, health, and demographic factors. We utilized two measures of sustainable working life: 1) employed at least two consecutive years (n = 21,348), without interruptions due to SA (>14 days), DP, or unemployment; 2) 22-years of sustainable working life, i.e., those who were employed all years from 1994 to 2016 (n = 12,931) without SA (>14 days), DP, or unemployment. Old-age pension, emigration, or death were censored. The final sample included same-sexed twin pairs of known zygosity; monozygotic pairs n = 11,403 and dizygotic pairs n = 13,354. Classical twin modelling was applied to estimate the relative contributions of genetic and environmental factors to individual differences in sustainable working life.

**Results:**

Individual differences for two consecutive years of sustainable working life were explained by genetics 36%, shared environment 8%, and unique environmental factors 56%. For 22-years of sustainable working life genetics accounted for 18%, shared environment 46%, and unique environmental factors for 37% of individual differences.

**Conclusions:**

Individual variation in sustainable working life is due to both genetic and environmental factors. Environmental experiences that twin siblings share while growing up is of importance, especially for long-term sustainable working life.

**Key messages:**

• Childhood environmental circumstances as well as environmental exposures later in life seem to outweigh genetic influences on long-term sustainable working life.

• From a public health perspective, the importance of family and surrounding environment on sustainable working life implies a potential for workplace or societal interventions, or individual support.

